# Adaption of the Memorial Sloan Kettering Cancer Center Nomograms for the Prediction of Prostate Cancer–specific Death in Sweden: A Population-based Cohort Study

**DOI:** 10.1016/j.euros.2025.06.003

**Published:** 2025-07-14

**Authors:** Renata Zelic, Marcus Westerberg, Pär Stattin, Hans Garmo, Lorenzo Richiardi, Olof Akre, Andreas Pettersson

**Affiliations:** a Department of Molecular Medicine and Surgery, Karolinska Institutet, Stockholm, Sweden; b Department of Pelvic Cancer, Cancer Theme, Karolinska University Hospital, Stockholm, Sweden; c Department of Surgical Sciences, Uppsala University, Uppsala, Sweden; d Cancer Epidemiology Unit, Department of Medical Sciences, University of Turin, Turin, Italy; e CPO-Piemonte, Turin, Italy; f Clinical Epidemiology Division, Department of Medicine Solna, Karolinska Institutet, Stockholm, Sweden

**Keywords:** Competing risk analysis, Nomogram, Other-cause mortality, Prognostic model, Prognostic model updating, Prostate cancer, Prostate cancer–specific mortality, Radical prostatectomy

## Abstract

**Background and objective:**

Prognostication is a cornerstone of the clinical management of prostate cancer. This study aims to update the pre- and postoperative Memorial Sloan Kettering Cancer Center (MSKCC) nomograms for the prediction of 10-yr prostate cancer–specific mortality in the competing risk setting in Sweden, and to evaluate the added value of comorbidities.

**Methods:**

A cohort study was conducted including all men in the National Prostate Cancer Register of Sweden diagnosed with localised prostate cancer in 2007–2020, who underwent radical prostatectomy. Follow-up was until December 31, 2022. We used cause-specific Cox proportional hazard models to obtain the cumulative incidence of prostate cancer–specific and other-cause mortality. The models were validated in terms of discrimination (concordance [C] index) and calibration by internal-external validation in six Swedish health care regions and by bootstrapping (*N* = 500).

**Key findings and limitations:**

The cohort included 31 106 men, of whom 629 died from prostate cancer and 2415 died from other causes during a median follow-up of 8.3 yr (interquartile range: 5.2, 11.8). Comorbidities added more value to the other-cause mortality model than to the prostate cancer–specific mortality model, and were included in all models. Both the preoperative and the postoperative model showed high discrimination for prostate cancer–specific death (optimism-corrected C-index: 0.81 and 0.87, respectively), but not for other-cause mortality (0.67, both models). All models were well calibrated, with minimal overestimation at the higher range of predicted cumulative incidences for the preoperative, but not for the postoperative, model.

**Conclusions and clinical implications:**

The updated MSKCC nomograms performed well in terms of discrimination and calibration, and can be used in clinical practice in Sweden. In this study, comorbidity added minimal prognostic value for predicting prostate cancer–specific mortality. External validation is advised for application in other populations.

**Patient summary:**

Prognostication is a cornerstone in the clinical management of prostate cancer. In this study, we adapted the best preforming risk classification system, the pre- and postoperative Memorial Sloan Kettering Cancer Center nomograms, for the prediction of prostate cancer–specific death in Swedish setting. The adapted models perform well and can be applied directly to Swedish men with prostate cancer.

## Take Home Message

The pre- and postoperative Memorial Sloan Kettering Cancer Center nomograms updated to the Swedish setting show high discrimination for prostate cancer–specific death at 10 yr. The models were well calibrated and can be used in clinical practice in Sweden.

## 1 Introduction

Prognostication is a cornerstone of the clinical management of prostate cancer given the wide range of prognosis. In our recent study [1], the Memorial Sloan Kettering Cancer Center (MSKCC) nomogram for biochemical recurrence after radical prostatectomy (RP) performed best in predicting prostate cancer death among Swedish men, outperforming other commonly used risk grouping systems such as the D’Amico and the National Comprehensive Cancer Network (NCCN) system. Although prostate cancer is typically slow progressing, comorbidity and competing mortality are not considered in most risk stratification tools.

Given the variation in diagnostic intensity and baseline risk between populations, validation and calibration of models based on local data are of paramount importance. Sweden has, for example, a substantially higher rate of prostate cancer–specific mortality than the USA, the country where the MSKCC models were developed, and many other countries [2]. In this study, we used the National Prostate Cancer Register (NPCR) of Sweden, a nationwide population-based cohort, to update the MSKCC nomogram to Swedish men who underwent primary RP. We accounted for competing events, evaluated the added value of comorbidities, and assessed the variation of the individual predicted 10-yr risks of prostate cancer–specific mortality across risk groups.

## 2 Patients and methods

### 2.1 Data sources

The NPCR is a clinical cancer register covering >95% of all incident cases of prostate cancer in Sweden since 1998 [3,4]. The NPCR aims to optimise the quality of care of men with prostate cancer by increasing adherence to national guidelines [5,6]. It includes comprehensive data on the cancer characteristics, diagnostic workup, and primary treatment.

Prostate Cancer data Base Sweden (PCBase) is a nationwide clinical research database constructed by linking the NPCR to other nationwide health care registers and demographic databases [3,4]. The National Patient Register includes all diagnostic and procedural International Classification of Diseases (ICD) codes from inpatient (since 1987) and outpatient (since 2001) care. The National Patient Register has extensively been validated for a number of diagnoses with a median positive predictive value of 84%, compared with patient chart data [7,8]. The Cause of Death Register contains the date and cause of death with close to 100% completeness since 1952 [9]. For prostate cancer, the agreement between the Cause of Death Register and medical record review is 86–96% [10,11]. The Prescribed Drug Register contains data on all drugs prescribed and dispensed in outpatient care in Sweden since 2005 [12]. The Total Population Register contains data on 100% of births and deaths, 95% of immigrations, and 91% of emigrations for all Swedish residents [13].

### 2.2 Study population

We included all men in the NPCR diagnosed in 2007–2020 with localised prostate cancer, defined as N0/Nx and M0/Mx at the preoperative workup, and treated with RP within 1 yr from diagnosis. The men were followed from the date of RP [14] until emigration, death, or December 31, 2022. A detailed description of the population selection is presented in Supplementary Fig. 1.

### 2.3 Outcome

The primary outcome was prostate cancer–specific death within 10 yr after RP, defined as prostate cancer listed as the underlying cause of death (ICD-10 code: C61) in the Cause of Death Register. The secondary outcome was 10-yr other-cause mortality.

### 2.4 Predictors

The predictors were selected to match the variables included in the pre- and postoperative MSKCC nomograms predicting biochemical recurrence (Supplementary Table 1) with some important distinctions. Briefly, in the preoperative model, the number of cores with and without cancer were modelled as continuous variables with a spike at 0 by including the type of biopsy (systematic/targeted) in the model [15]. Clinical tumour stage was grouped as cT1, cT2, and cT3+, and we included the calendar year and the time between diagnosis and RP into the model. In the postoperative model, pathological tumour stage was modelled as a categorical variable including all available stages (pT2, pT3, pT3a, pT3b, and pT4). As for the preoperative model, the type of biopsy, calendar year, and time to RP were included in the model. These distinctions and the reasoning behind them are explained in more detail in Supplementary Table 2.

Finally, to evaluate the added value of comorbidities, ischaemic heart disease, congestive heart failure, cardiac arrhythmia, hypertension, chronic obstructive pulmonary disease, diabetes mellitus, and other tumours were included in the models as binary indicators (Supplementary Table 2). The selected comorbidities were either most common of the available comorbidities in the PCBase or deemed important based on subject-specific knowledge. The definitions of the comorbidities are available in Supplementary Table 3.

### 2.5 Statistical analyses

#### 2.5.1 Selection of models and functional forms

All prespecified predictors (Supplementary Table 2) were included in the models. We used fractional polynomial-based procedure to select functional forms for continuous variables [[15], [16], [17]] and evaluated the stability of the selected form by bootstrapping (Supplementary Table 4 and Supplementary Fig. 2) [18,19]. Functional forms for age (power 1, ie, no transformation), prostate-specific antigen (PSA; power 0, ie, $ln\left[PSA\right)]$), the number of cores with (power 1) and without (power 1) cancer, and time to RP (power 1) were selected in the preoperative model population. The same functional forms for age, PSA, and time to RP were used for the postoperative model.

The added value of comorbidities was evaluated by the following: (1) explained variability [20,21], (2) likelihood ratio test comparing the models with and without comorbidities, and (3) plotting the distribution of the 10-yr risks predicted from the model with and without comorbidities.

#### 2.5.2 Model development

Men with missing predictor information (3.4% in the preoperative and 5.0% in the postoperative model population) were excluded from the analyses (Supplementary Fig. 1). The models in this study have been updated from the original MSKCC models in several ways. First, these predict different outcomes. Second, we have used different parameterisations for some of the predictors. Third, we have added new predictors. As a result, all model coefficients and baseline hazards had to be re-estimated, and may not be comparable with those in the original MSKCC models. To account for the presence of competing events/other-cause mortality, we used cause-specific Cox proportional hazard models to re-estimate model coefficients and to estimate the cause-specific hazards of cancer-specific and other-cause mortality [22]. The included men were followed from the date of RP and censored at the date of death (the event of interest or the competing event), emigration, or at the end of follow-up (December 31, 2022), whichever came first. The two cause-specific hazards were combined to obtain cumulative incidence functions (CIFs) for each event and each patient.

#### 2.5.3 Model validation

We performed internal-external validation by cross-validation, where the six Swedish health care regions were used as cross-validation folds, and internal validation by bootstrapping [23,24]. All model development steps were repeated in each cross-validation and in 500 bootstrap samples. Model discrimination was evaluated by calculating the apparent, cross-validated, and optimism-corrected concordance (C) index [22,25], and calibration was done by plotting nonparametric CIFs estimated using the Aalen-Johansen estimator [26] and population-averaged, predicted CIFs over follow-up time, and by plotting observed and predicted 10-yr risks [[27], [28], [29]].

We further used bootstrapping to obtain uniform shrinkage factors [30,31] and re-estimate baseline hazards while keeping shrunk coefficients fixed [30]. The combination of the baseline hazards (Supplementary material) and coefficients for both events allows for full external validation of our models.

## 3 Results

The total study population included 31 106 men diagnosed with localised prostate cancer during 2007–2020, who underwent RP within 1 yr of diagnosis (Supplementary Fig. 1 and Table 1).

**Table 1:** Baseline characteristics of men in the National Prostate Cancer Register of Sweden diagnosed with localised prostate cancer during 2007–2020 who underwent radical prostatectomy within 1 yr from the date of diagnosis

	Study population (*n* = 31 106)
Age at diagnosis, median (IQR)	64 (60, 68)
Calendar year of RP, *n* (%)	
2007–2011	10 374 (33)
2012–2016	11 820 (38)
2017–2020	8912 (29)
Time to RP (d), median (IQR)	103 (74, 144)
Preoperative PSA (ng/ml), median (IQR)	6.6 (4.7, 10.0)
Missing, *n* (%)	166 (0.30)
Preoperative clinical tumour stage,^a^*n* (%)	
cT1	18 917 (61)
cT2	10 761 (35)
cT3	955 (3.1)
cT4	10 (0.03)
Missing	386 (1.2)
Type of biopsy,^b^*n* (%)	
Systematic	28 464 (92)
Targeted	2312 (7.4)
Missing	330 (1.1)
Number of cores with cancer, median (IQR)	3 (2, 5)
Missing, *n* (%)	650 (2.1)
Number of cores without cancer, median (IQR)	6 (4, 8)
Missing, *n* (%)	654 (2.1)
Preoperative biopsy Gleason score,^b^*n* (%)	
6	9413 (30)
3 + 4	13 366 (43)
4 + 3	4869 (16)
8	2202 (7.1)
9–10	1141 (3.7)
Missing	115 (0.37)
Pathological tumour stage,^c^*n* (%)	
pT2	19 690 (63)
pT3	1352 (4.4)
pT3a	6743 (22)
pT3b	2459 (7.9)
pT4	69 (0.22)
Missing	724 (2.3)
Pathological Gleason score,^b^*n* (%)	
6	6348 (20)
3 + 4	14 144 (45)
4 + 3	6830 (22)
8	1863 (6.0)
9–10	1571 (5.1)
Missing	350 (1.1)
Lymph node dissection,^b^*n* (%)	
Not performed	24 743 (80)
pN0	5132 (17)
pN1	895 (2.9)
Missing	336 (1.1)
Surgical margin status,^b^*n* (%)	
Negative	21 122 (68)
Positive	7753 (25)
Unclear	1711 (5.5)
Missing	520 (1.7)
Preoperative comorbidities, *n* (%)	
Hypertension	11 592 (37)
Diabetes mellitus	2487 (8.0)
Ischaemic heart disease	2057 (6.6)
Cardiac arrhythmia	2264 (7.3)
Congestive heart failure	414 (1.3)
Chronic obstructive pulmonary disease	451 (1.5)
Other cancer	1854 (6.0)

cT = clinical tumour stage; IQR = interquartile range; PSA = prostate-specific antigen; pT = pathological tumour stage; RP = radical prostatectomy.

a Percentages do not sum up to 100% because men with cT0 (*N* = 77, 0.25%) are not presented and because of rounding; cT0 was grouped with cT1 in the analyses.

b Percentages do not sum up to 100% due to rounding.

c Percentages do not sum up to 100% because men with pT0 (*N* = 69, 0.22%) are not presented and because of rounding. Men with pT0 were excluded from the analyses.

### 3.1 Added value of comorbidities

Of all the included men, 47% had at least one of the included comorbidities, of whom 31% had only one and 12% two concomitant comorbidities. Addition of comorbidities to the pre- and postoperative prostate cancer–specific mortality models had minimal impact on the explained variability and the spread of the predicted 10-yr risks (Supplementary Figs. 3 and 4). However, the likelihood ratio test indicated improvement of the preoperative model (Supplementary Table 5). Addition of comorbidities to the pre- and postoperative other-cause mortality models improved both models in terms of explained variability, likelihood ratio test (Supplementary Table 5), and the spread of predicted 10-yr risks (Supplementary Figs. 3 and 4). Though the added value for the prostate cancer–specific mortality model was not as clear, comorbidities were included in the final models for both outcomes.

### 3.2 Preoperative models

The preoperative model population included 29 985 men (Supplementary Table 6). The model coefficients are presented in Supplementary Table 7. The bootstrap-based uniform shrinkage factors were 0.94 and 0.98 for the models for cancer-specific and other-cause mortality, respectively (Supplementary Fig. 5).

**Fig. 1:**
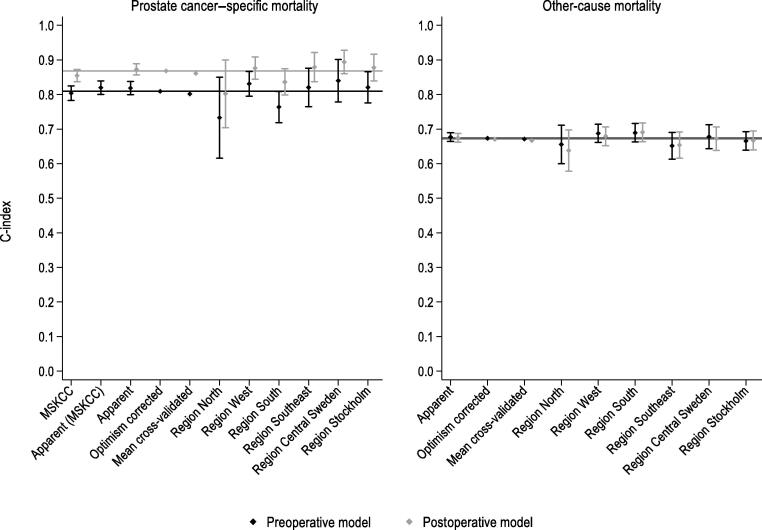
Discrimination of the pre- and postoperative models predicting prostate cancer–specific and other-cause mortality at 10 yr. Vertical lines present 95% confidence intervals for C-index estimates. Horizontal lines present the optimism-corrected C-index for the pre- and postoperative models. The cT2 and cT3 substages, which are included in the preoperative MSKCC nomogram but are not recorded in the National Prostate Cancer Register of Sweden, were calculated using models developed in the study of Zelic et al [1]. Apparent (MSKCC) refers to the preoperative model discrimination in the population with nonmissing variables needed for the calculation of cT2 and cT3 substages (N = 29 259). MSKCC = Memorial Sloan Kettering Cancer Center.

**Fig. 2:**
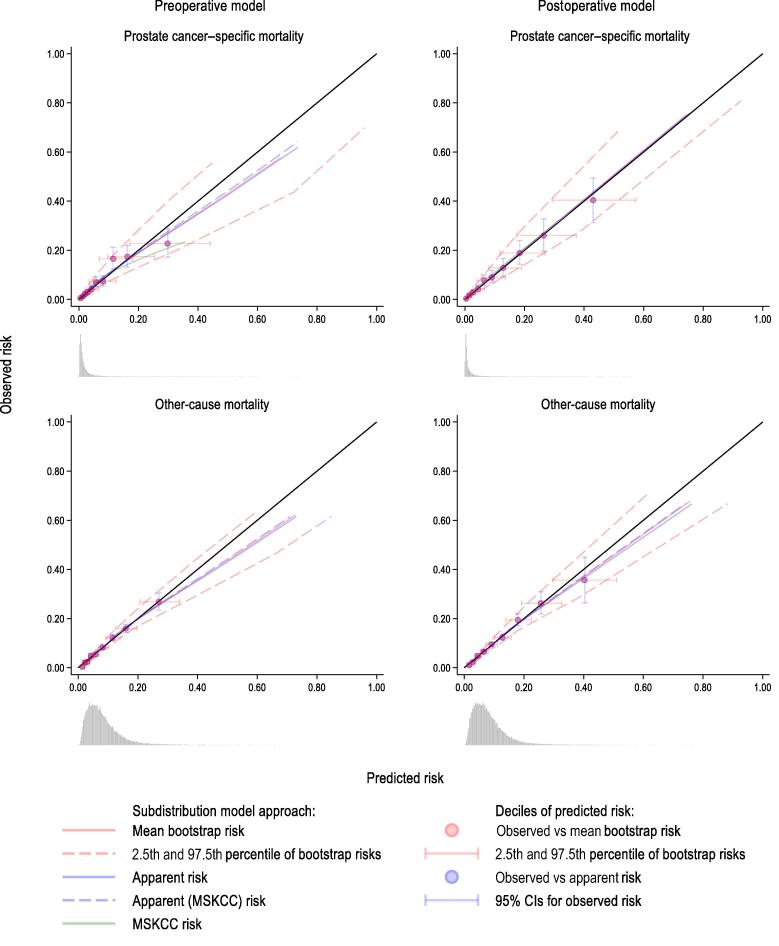
Calibration plot for the pre- and postoperative models predicting prostate cancer–specific and other-cause mortality at 10 yr. The apparent predicted risk is a risk predicted for the study population using estimates from the model developed using the study population. The mean bootstrap risk and 2.5th and 97.5th percentiles of bootstrap risks are calculated from 500 risks predicted for the study population using estimates from the models developed using the 500 bootstrap samples. Smooth calibration curves were created by modelling the outcome of interest as a function of the predicted risk transformed as log(-log[1-risk]) in a Fine and Gray model. The predicted risks were modelled using restricted cubic splines with four degrees of freedom. The predictions from this model were used as the observed outcome proportions. For each of 500 bootstraps, we averaged the estimated 10-yr risks in the groups defined by the deciles of the apparent predicted risk among individuals who had the event of interest, and plotted their mean and 2.5th and 97.5th percentiles against the Aalen-Johansson estimate. The cT2 and cT3 substages, which are included in the preoperative MSKCC nomogram but are not recorded in the National Prostate Cancer Register of Sweden, were calculated using models developed in the study of Zelic et al [1]. The apparent (MSKCC) risk refers to the apparent risk predicted by the preoperative model for the population that had nonmissing variables needed for the calculation of cT2 and cT3 substages (N = 29 259). A histogram plots the density of the apparent predicted risks. CI = confidence interval; MSKCC = Memorial Sloan Kettering Cancer Center.

The optimism-corrected and the average cross-validated C-indices were 0.81 and 0.80, respectively, for predicting the 10-yr prostate cancer–specific mortality, while these were both 0.67 for predicting the 10-yr other-cause mortality (Fig. 1 and Supplementary Table 8). The population-averaged apparent CIFs were very similar to the observed CIFs, indicating overall good calibration, while a complete overlap between the apparent and the mean bootstrap CIF indicated no optimism in the apparent estimates (Supplementary Fig. 6). There was, however, minimal underestimation of CIFs for prostate cancer–specific mortality after 6 yr of follow-up (Supplementary Fig. 6). The underestimation of CIFs for prostate cancer–specific mortality was more pronounced in the West health care region, and that of CIFs for other-cause mortality was more pronounced in the South region (Supplementary Fig. 7). When we evaluated calibration of the 10-yr risks in the groups defined by the deciles of the predicted risk and via smoothed calibration plots, we observed no optimism in the apparent estimates, but there was some overestimation at the higher range of predicted risks (Fig. 2).

Compared with the original model, updating of the preoperative MSKCC nomogram had minimal impact on model discrimination in Swedish population (C-index 0.80 vs 0.81; Fig. 1 and Supplementary Table 8), but it improved calibration of population-averaged CIFs at all times as well as the calibration and spread of the predicted 10-yr risk (Fig. 2, and Supplementary Figs. 6 and 8).

### 3.3 Postoperative models

The postoperative model population included 29 470 men (Supplementary Table 9). The model coefficients are presented in Supplementary Table 10. The bootstrap-based uniform shrinkage was 0.97 for both the cancer-specific and the other-cause mortality model (Supplementary Fig. 5).

The postoperative model discriminated death from prostate cancer at 10 yr better than the preoperative model, with optimism-corrected C-index of 0.87 and cross-validated C-index of 0.86 (Fig. 1 and Supplementary Table 8). Discrimination of the postoperative model predicting other-cause mortality was practically unchanged compared with that of the preoperative model (Fig. 1). The population-averaged apparent CIFs were similar to the average observed CIFs over the entire follow-up time, with no optimism in the apparent estimates (Supplementary Fig. 6). The discrepancies between the observed and predicted CIFs were similar to those of the preoperative model (Supplementary Figs. 6 and 9). When we evaluated calibration of the 10-yr risks, we observed no optimism in the apparent risk estimates; however, there was some overestimation at the higher range of the predicted other-cause mortality risks (Fig. 2).

As for the preoperative model, updating of the postoperative MSKCC nomogram improved the calibration of population-averaged CIFs and the spread of the predicted 10-yr risk (Supplementary Table 8, Fig. 1, Fig. 2, and Supplementary Figs. 6 and 8).

### 3.4 Individual predicted risks of prostate cancer–specific death by risk group

**Fig. 3:**
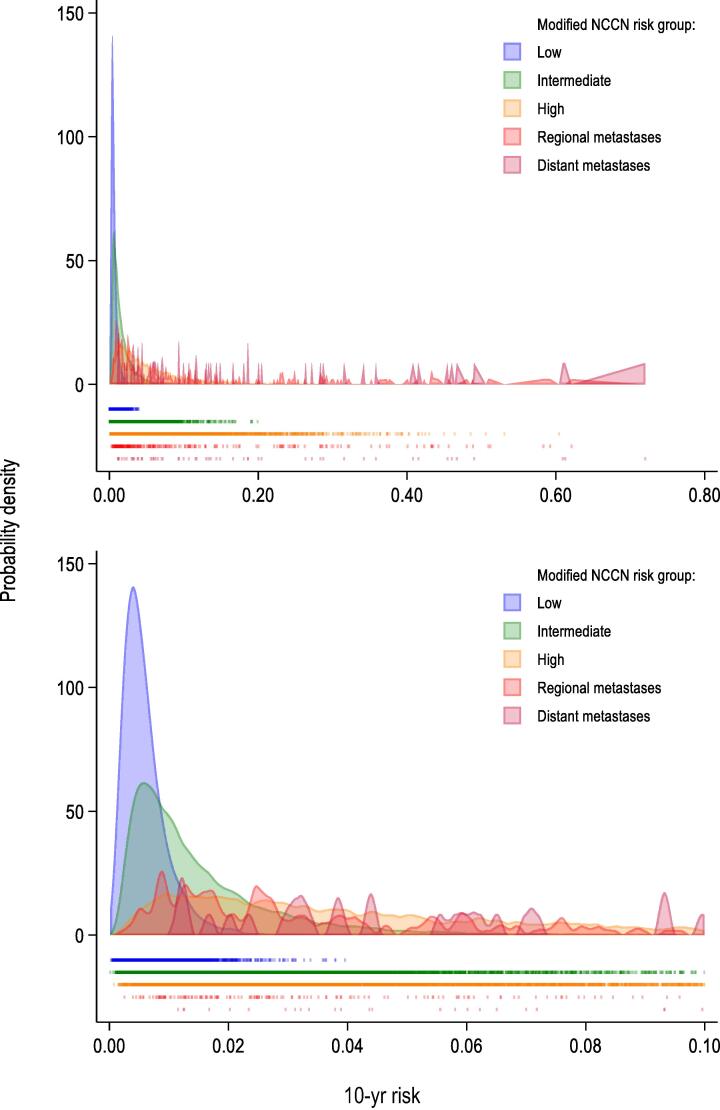
The distribution of the preoperative model–predicted 10-yr risk of prostate cancer–specific mortality in the levels of modified NCCN risk groups. Risk groups were defined as follows: low risk: cT1–2 and Gleason score ≤6 and PSA <10; intermediate risk: not cT3–4 and Gleason score 7 and PSA <20, or not cT3–4 and Gleason score ≤6 and 10 ≤ PSA < 20; high risk: cT3 and any Gleason score and PSA <50, or not cT4 and Gleason score 8–10 and PSA <50, or not cT4 and any Gleason score and 20 ≤ PSA < 50; regional metastases: PSA ≤100 and N1, or cT4 and PSA ≤100, or 50 ≤ PSA  ≤ 100; and distant metastases: PSA >100. NCCN = National Comprehensive Cancer Network; PSA = prostate-specific antigen.

The distribution of predicted 10-yr risk of prostate cancer–specific death by modified NCCN risk group [4,32] is shown in Fig. 3, and the respective results by Swedish guidelines risk groups [33], NCCN risk groups [34], and CAPRA score [35] in Supplementary Fig. 10. The individual 10-yr risks varied from 0.03% to 4.0% (median risk: 0.51%), 0.01% to 20% (median risk: 1.1%), and 0.08% to 60% (median risk: 4.0%) within the NCCN low-, intermediate-, and high-risk groups, respectively. For example, a 66-yr-old man with an intermediate-risk cancer, cT2, Gleason score 4 + 3, and PSA 0.5, who had 1/12 systematic biopsy cores with cancer, had a 10-yr risk of prostate cancer death of 0.47%. The corresponding risk was 19% for a 68-yr-old man with an intermediate-risk cancer, cT2, Gleason score 4 + 3, PSA 11, and 12/12 systematic biopsy cores with cancer.

## 4 Discussion

In this nationwide population-based study, we adapted the MSKCC nomograms to Swedish men with localised prostate cancer who underwent RP. We further refined the models by accounting for competing events and by including comorbidity data in the models. Both the preoperative (C-index 0.81) and the postoperative (C-index 0.87) model performed well in terms of discriminating prostate cancer–specific death at 10 yr and were well calibrated. Compared with the original MSKCC nomogram, updating had minimal impact on model discrimination, but it improved calibration for the preoperative model and led to a much wider range of predicted individual risks for both the preoperative and the postoperative model. We also observed strong variations of individual 10-yr risks of prostate cancer–specific mortality across risk groups: 0.03–4.0%, 0.01–20%, and 0.08–60% for the modified NCCN low-, intermediate-, and high-risk groups, respectively.

For all models, that is, the pre- and postoperative models for both outcomes, the model performance was similar using different approaches of model validation, that is, internal-external cross-validation and bootstrap validation, indicating that the models are robust. All models were well calibrated with no optimism in the apparent estimates. We, however, observed some overestimation at the higher range of predicted risks for the preoperative model. While this overestimation should be considered when applying the models to men with a particularly high predicted risk, it is unlikely to cause practical changes in the clinical decision-making.

Discrimination for prostate cancer–specific death at 10 yr was high, with a C-index of 0.81 for the preoperative model and 0.87 for the postoperative model. Addition of comorbidities improved model fit; however, it did not add much value to these models in terms of improvement in explained variation, discrimination, or range of predicted risks, which is in line with the findings of most [[36], [37], [38], [39], [40], [41], [42]], though not all [43,44], prior studies assessing comorbidities as independent predictors of prostate cancer death. At the same time, the value added by comorbidities to the other-cause mortality model was more obvious, suggesting that our comorbidity data capture comorbidity burden in a relevant way. It is possible that a more extensive list of comorbidities could further improve the prediction of prostate cancer–specific mortality.

Discrimination of our other-cause mortality model was lower than that of the prostate cancer–specific mortality model (C-index 0.67 for both the preoperative and the postoperative model). Even though estimated life expectancy is important in prostate cancer management [45,46], there are no clear recommendations regarding which life-expectancy model to use. The original MSKCC models predicting biochemical recurrence and prostate cancer–specific mortality after RP are not intended for prediction of other- or all-cause mortality. Although the MSKCC has also developed a life-expectancy nomogram for men with untreated prostate cancer, it is unclear how this nomogram generalises to men who underwent RP. By publishing the other-cause mortality models in addition to the cause-specific mortality models, we aim at facilitating informed clinical decision-making for men treated with RP. While incorporation of comorbidity data added value to our model and made it more informative for clinical decision-making compared with the commonly used age-adjusted life tables, development of an extensive other-cause mortality model was beyond the scope of this paper. As demonstrated in other studies, discrimination of the other-cause mortality model can be improved by, for example, the inclusion of an extensive list of comorbidities/comorbidity indices and socioeconomic proxies, and by modelling complex interactions [42,[47], [48], [49], [50], [51]]. Such complex models are, however, difficult to use clinically unless implemented in electronic health records, and the extent of improvement in individual risk prediction compared with simpler models is unclear.

We observed substantial heterogeneity of prostate cancer–specific mortality risk within the established risk groups. In the intermediate-risk group, for example, the risk varied from 0.01% to 20%. A practical way to incorporate these findings into clinical practice is to use information on standard risk groups plus individual predicted risks. Combining information on risk groups with individual risk assessments can improve clinical decision-making. That said, when using the predictions from the preoperative models to make treatment choices, it is important to consider that the absolute risk estimates are based on outcomes following RP. A web-based risk calculator based on the findings of this study that can be used in clinical practice in Sweden is available at: https://npcr.se/nomograms/.

These models are meant to be used for Swedish men with N0/Nx and M0/Mx prostate cancer who were planned for and have undergone RP. To be applied to men treated with other modalities or to populations with a different distribution of predictors and/or outcomes (ie, to populations with other prostate cancer characterises, prostate cancer mortality rates, etc.), the models should be first validated in such populations and updated if performance at external validation is not satisfactory.

While our population is relatively contemporary, information on targeted biopsy–related predictors are limited in the NPCR. Our models can still be applied to men who underwent a targeted biopsy, but the predicted risk will be less granular since the number of biopsies with/without cancer was not used for this group. However, we intend to dynamically update these models over time when such information becomes available. A large proportion of the study population has low- or intermediate-risk disease (see Table 1), reflecting historical treatment patterns. Many such patients are today undergoing active surveillance rather than RP. During the study period, the use of active surveillance in Sweden increased twofold [52]. However, our models should still be relevant for men with high-risk disease who are similar to the high-risk men in the study population. Finally, to increase completeness of information on comorbidities, we used records from the Inpatient and Outpatient Register up until and including the date of RP, as it is more likely that serious comorbidities will also be recorded as a part of a preoperative assessment. Where necessary, we also used information on the prescribed drugs, that is, for hypertension and diabetes mellitus. Nevertheless, some misclassification of the comorbidity information may still be possible for comorbidities not requiring inpatient/outpatient visit or treatment.

## 5 Conclusions

In conclusion, the pre- and postoperative MSKCC nomograms adapted to the Swedish setting show high discrimination for prostate cancer–specific death at 10 yr. The models were well calibrated and can be used in clinical practice in Sweden. External validation of the current or original MSKCC nomogram is recommended for use of the models in other populations.

  ***Author contributions:*** Renata Zelic had full access to all the data in the study and takes responsibility for the integrity of the data and the accuracy of the data analysis.

  *Study concept and design*: Zelic, Pettersson, Akre, Richiardi.

*Acquisition of data*: Westerberg, Statin, Garmo.

*Analysis and interpretation of data*: Zelic, Westerberg, Pettersson, Richiardi, Akre, Garmo, Statin.

*Drafting of the manuscript*: Zelic, Pettersson.

*Critical revision of the manuscript for important intellectual content*: Zelic, Pettersson, Akre, Richiardi, Westerberg, Garmo, Statin.

*Statistical analysis*: Zelic.

*Obtaining funding*: Akre, Richiardi.

*Administrative, technical, or material support*: Pettersson, Akre, Westerberg, Statin, Garmo.

*Supervision*: Pettersson, Akre, Richiardi.

*Other*: None.

  ***Financial disclosures:*** Renata Zelic certifies that all conflicts of interest, including specific financial interests and relationships and affiliations relevant to the subject matter or materials discussed in the manuscript (eg, employment/affiliation, grants or funding, consultancies, honoraria, stock ownership or options, expert testimony, royalties, or patents filed, received, or pending), are the following: None.

  ***Funding/Support and role of the sponsor:*** This work was supported by the Cancerfonden (23 3256 S 01 H; 22 2324 Pj 01 H), Prostatacancerförbundet, Radiumhemmets Forskningsfonder, ITEA-Vinnova (Symphony; 2022-01275) and the Italian Association for Cancer Research.. The funding organisations had no role in the design and conduct of the study; collection, management, analysis, and interpretation of the data; preparation, review, or approval of the manuscript; and decision to submit the manuscript for publication.

  ***Data sharing statement:*** Data that support the findings of this study are registry data of patients providing routinely collected data and are not available to other researchers. However, anyone with an appropriate ethical committee approval can apply for access to the data.

  ***Acknowledgements:*** Collection of data in the NPCR of Sweden was made possible by the continuous work of the NPCR steering group: Elin Axén, Johan Styrke, Andreas Josefsson, Camilla Thellenberg, Hampus Nugin, Ingrida Verbiené, Stefan Carlsson, Anna Kristiansen, Mats Andén, Kimia Kohestani, Jon Kindblom, Thomas Jiborn, Olof Ståhl, Olof Akre, Eva Johansson, Magnus Törnblom, Fredrik Jäderling, Marie Hjälm-Eriksson, Lotta Renström Koskela, Erik Thimansson, Johan Stranne, Elin Trägårdh, Viktoria Gaspar, Fredrik Sandin, Petrus Stenson, Lena Pettersson, Mia Brus, Gustaf Hedström, Anna Hedström, Maria Moutran, Nina Hageman, and Maria Nyberg, and patient representatives Hans Joelsson and Gert Malmberg.
